# RUBic: rapid unsupervised biclustering

**DOI:** 10.1186/s12859-023-05534-3

**Published:** 2023-11-16

**Authors:** Brijesh K. Sriwastava, Anup Kumar Halder, Subhadip Basu, Tapabrata Chakraborti

**Affiliations:** 1 Computer Science and Engineering Department, Government College of Engineering and Leather Technology, Kolkata, India; 2 Faculty of Mathematics and Information Sciences, Warsaw University of Technology, Warsaw, Poland; 3 CeNT, University of Warsaw, Warsaw, Poland; 4 Department of Computer Science and Engineering, Jadavpur University, Kolkata, 700032 India; 5 The Alan Turing Institute and University College London, London, UK

**Keywords:** Data mining, Algorithm design and analysis, Biclustering algorithms, Computational complexity

## Abstract

Biclustering of biologically meaningful binary information is essential in many applications related to drug discovery, like protein–protein interactions and gene expressions. However, for robust performance in recently emerging large health datasets, it is important for new biclustering algorithms to be scalable and fast. We present a rapid unsupervised biclustering (RUBic) algorithm that achieves this objective with a novel encoding and search strategy. RUBic significantly reduces the computational overhead on both synthetic and experimental datasets shows significant computational benefits, with respect to several *state-of-the-art* biclustering algorithms. In 100 synthetic binary datasets, our method took $\sim 71.1$ s to extract 494,872 biclusters. In the human PPI database of size $4085\times 4085$, our method generates 1840 biclusters in $\sim 48.6$ s. On a central nervous system embryonic tumor gene expression dataset of size 712,940, our algorithm takes   101 min to produce 747,069 biclusters, while the recent competing algorithms take significantly more time to produce the same result. RUBic is also evaluated on five different gene expression datasets and shows significant speed-up in execution time with respect to existing approaches to extract significant KEGG-enriched bi-clustering. RUBic can operate on two modes, base and flex, where base mode generates maximal biclusters and flex mode generates less number of clusters and faster based on their biological significance with respect to KEGG pathways. The code is available at (https://github.com/CMATERJU-BIOINFO/RUBic) for academic use only.

## Introduction

Specialised gene clusters participate in specific cellular processes under a subset of conditions. Automatic identification of such gene-clusters and condition-subsets is usually known as biclustering and has immense value in such applications of bioinformatics (proteomics and genomics) like studying protein–protein interactions, automated drug discovery. The concept of biclustering algorithms was first introduced by Hartigan et al. [1]. Cheng et al. [2] first applied biclustering of both genes and condition-subsets for knowledge discovery from expression data. A node-deletion algorithm was presented by Cheng et al. [2] to find bicluster in expression data that have low mean squared residue scores. It found similarity based on a subset of attributes, clustering of genes and conditions, and overlapped bicluster that provides a better representation for genes with multiple functions or regulated by many factors. Their method allowed discovery of similar genes based on a subset of attributes, i.e., simultaneous clustering of genes and conditions. Subsequently, biclustering techniques have been widely applied in gene expression data analysis. In contrast to biclustering algorithms, such as the recently developed graph clustering algorithms [3–5], which can be applied to complex biological datasets, they have constraints related to unannotated data and dataset size.

In the field of biclustering within the realm of bioinformatics, recent years have seen the rise of numerous advanced computational methods. Many of these methods leverage the capabilities of machine learning. These approaches can be broadly categorized into two main groups: graph-based and non-graph-based biclustering techniques. Moreover, it’s worth noting that within these categories, individual algorithms are further subdivided based on their compatibility with different types of input data, with some designed to operate on raw expression data and others tailored for binary data. Tanay et al. [6] proposed a method that combines graph theory with statistical modeling and has polynomial-time complexity. Yang et al. [7] presented a probabilistic algorithm called FLOC for finding k possibly overlapping biclusters used graph theory to address the order-preserving submatrix problem by identifying submatrices that preserve order relationships among genes and conditions. Other graph based approaches are ISA [8, 9], Samba [6], OPSM [10] , spectral biclustering [11], spectral biclustering [11] and xMotif [12] where xMotif utilizes graph theory to identify conserved gene expression motifs by representing genes and conditions as nodes in a graph and finding significant subgraphs that correspond to biclusters.

Some of the non-graph based biclustering methods introduced greedy heuristic idea or machine learning. Cheng et al. [13] proposed a greedy version of an existing biclustering algorithm. Santamaria et al. [14] developed BicOver-lapper for visualizing biclusters from gene-expression matrices, whereas Uitert et al. [15] designed an algorithm to extract biclusters from sparse, binary datasets. Madeira et al. [16] presented the ‘e-CCC-Biclustering’ algorithm that mines coherent biclusters with approximate expression patterns. The FABIA tool [17] uses a multiplicative model and Bayesian techniques, whereas the DeBi algorithm [18] uses a frequent item set-based data mining approach to determine homogeneous biclusters. Sill et al. [19] offered a sparse singular value decomposition (SSVD) approach to control Type I error rates and discover stable biclusters. Huang et al. [20] presented a biclustering method based on evolutionary learning and applied it in a search space created by the conditions. Ayadi et al. [21] introduced a heuristic algorithm, ‘BicFinder’, to estimate the coherence of a given bicluster. Huang et al. [22] proposed biclustering for mining to find technical trading patterns that combine indicators from historical financial data series. Prelic et al. [23] proposed a fast and exact model biclustering algorithm called Bimax, considered a benchmark reference for most biclustering methods. Bimax achieved similar scores as the best biclustering techniques then and is still helpful in identifying potentially relevant ground-truth biclusters as a pre-processing step. Later, these chosen biclusters can be used as input for more accurate biclustering methods to speed up processing time and increase bicluster quality.

One common challenge faced by many of these biclustering algorithms is their inability to guarantee finding the global optima, potentially leading to the omission of some optimal biclusters. An algorithm known for its benchmark status in this regard is Bimax, introduced by Prelic et al. [23]. Bimax, designed for binary data models, has exhibited substantial performance improvements compared to other biclustering methods, including ISA [8, 9], Samba [6], OPSM [10], CC [2], and xMotif [12]. However, xMotif tend to identify large biclusters representing gene groups with stable expression levels, potentially lacking interesting patterns such as co-regulation. ISA, Samba, and OPSM have demonstrated comparable performance, with Samba excelling in managing increased regulatory complexity but being more sensitive to noise than ISA. Bimax serves as a valuable preprocessing step to identify potentially relevant ground-truth biclusters, which can then be utilized as input for more accurate biclustering methods to enhance processing speed and bicluster quality. Although initially designed for binary data, Bimax has been extended to handle real-valued data, leading to the development of BiBit [24] and its successor, QUBIC [25], and QUBIC2 [26]. BiBit, an extension of Bimax, introduces a preprocessing step to transform data into binary format. QUBIC2, in contrast, employs a probabilistic model to directly handle real-valued data, showcasing superior accuracy and efficiency compared to BiBit and other state-of-the-art biclustering algorithms on both synthetic and real datasets. A more recent entrant, ARBic [27], outperforms QUBIC and QUBIC2 in discovering high-quality biclusters with varying dimensions and shapes while claiming increased robustness to noise and outliers. Both QUBIC2 and ARBIC algorithms involve essentially a two-step procedure: initial seed point selection and subsequent expansion to the biclustering, separated into 2 separate stages. The seed selection process for QUBIC2 and ARBIC relies on additional graph creation from all data points, leading to increased computational demands.

In this work, we present the Rapid Unsupervised Biclustering (RUBic) algorithm which demonstrates significantly enhanced speed in comparison to recently developed approaches, while effectively extracting all original biclusters from both synthetic and biological datasets. The aim of reducing computational overhead with respect to *state-of-the-art* algorithms [24, 26, 27] was achieved by introducing a novel encoding and search strategy. The methodology presented in this article necessitates significantly fewer computational steps compared to BiBit [24], while producing the same maximal biclusters within a notably reduced timeframe, denoted as the base mode. Additionally, through the utilization of flex-mode tuning in RUBic, biologically significant clusters were generated, surpassing those obtained by the most recently developed ARBic [27] and QUBIC2 [26] algorithms, as confirmed by KEGG pathway [28] annotations. Thus the contribution of this work is two-fold:

1. A new biclustering algorithm (RUBic) that is significantly faster with reduced computational load (shown both mathematically and experimentally) than the recent competitors and hence establishing a new state-of-the-art that has great potential for robust scalability in large healthcare datasets.
2. Unlike existing methods, RUBic can operate in two modes, the default mode produces the maximal set of biclusters like its competitors but faster, and a flex mode where conditional priors can be provided for biologically relevant subsets (validated with KEGG pathways) and hence has great potential in genomics/proteomics applications like automated drug discovery.

## Methods

The developed RUBic method is designed to extract maximal biclusters from binary datasets. It takes input parameters as the binary input matrix (*A*) as well as the minimum number of rows $(r_{min})$ and columns $(c_{min})$ which are allowed in the final biclusters. Let the input binary matrix be $A=(R,C)$, where *R* and *C* are two finite sets of rows and columns, respectively. Let $l:R \times C \rightarrow {\{0,1\}}$ be a binary function for the matrix *A*. Let the binary value $l(r,c); r \in R,c \in C$, be denoted by $\{b_{rc}\}$ and the corresponding decimal value be represented by $[[d_{rc}]], d_{rc} \in D; D={\{0,\ldots ,15\}}.$The binary matrix $A=(R,C)$, can be decomposed into *N* sets of *M* bits, such that $A={\{r_1,\ldots ,r_N\}},$ with $r_i={\{b_{i1},\ldots ,b_{iM}\}},$ where $b_{ij} \in \{0,1\}, N =|R|$ and $M=|C|.$

**Fig. 1:**
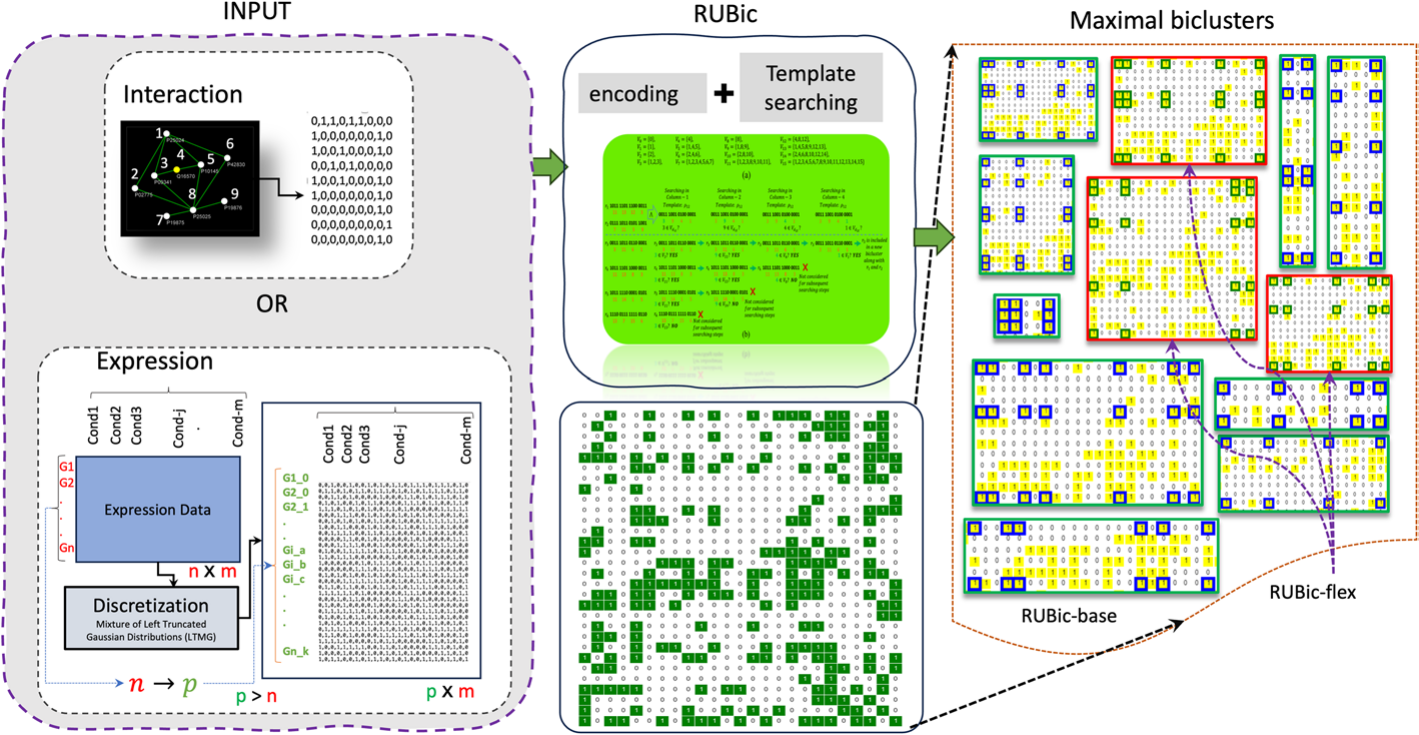
Basic workflow of RUBic. The unsupervised biclustering strategy works both in interaction data and expression data. Initially, it converts the expressions into binary data using mixture of left truncated Gaussian distribution model (LTMG) and find the biclusters using novel encoding and template searching strategy and finally generates the biclusters in two modes base and flex. In base mode RUBic generates maximal biclusters (green borders) and in flex mode results less and biological significant clusters (red bordered). Coloured cell box within the clusters indicates the selected row and column positions

Let, $B=\{B_1,\ldots ,B_k\}$ be the set of biclusters, such that $B_i=\{I_i,J_i\}$, is composed of the pair of non-empty sets, where $I_i \subseteq R$ and $J_i \subseteq C$. For any given bicluster $B_i$, a set of columns $J_i =\{c_1,\ldots ,c_K\}$ is called a template if for every $c_k \in J_i$ and for every pair of rows $(r,r^{'}) \in I_i$, we have $\{r,c_{k} \} \bigwedge \{r',c_{k}\}=1$. The bicluster $B_i=\{I_i,J_i\}$ is called a maximal bicluster if and only if it is not entirely contained in any other bicluster. In another way, the biclusters will be maximal sub-matrices created from a template obtained by the application of the bit-wise AND operator $(\bigwedge )$ to a pair of seed rows. Now, the problem addressed by the developed biclustering algorithm can be formally defined as follows: given a binary data matrix *A*, we want to identify a set of biclusters $B=\{B_1,\ldots ,B_k\}$, where, $B_i=(I_i,J_i)$ such that each bicluster $B_i$ satisfies some particular features of homogeneity. Note that, the exact characteristics of homogeneity that a bicluster follow may differ from one approach to another approach. In our work, the homogeneity characteristic is governed by: (1) encoding of the *template* from a pair of seed rows by bit-wise AND operations, and (2) efficient searching of the remaining rows, having common homogeneity characteristic, for possible inclusion in the bicluster. The overall working principle of RUBic is depicted in Fig. 1.

### Encoding strategy

**Fig. 2:**
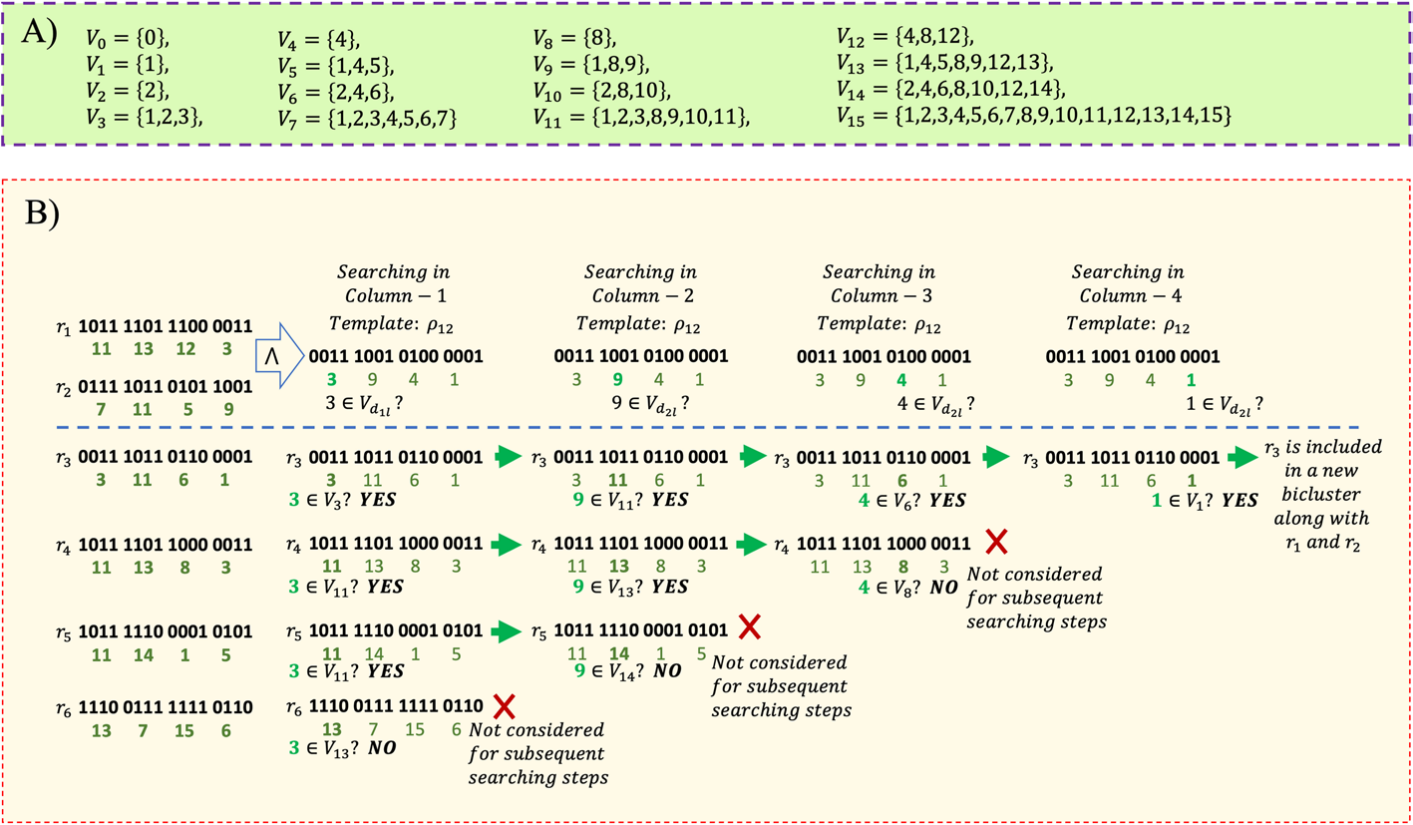
The underlying concepts of the encoding and the searching strategy of the RUBic algorithm are illustrated with the help of an example; **A** the composition of the set $V=\{V_{i}\}, \forall i \in D$, and **B** the steps involved in formation of a bicluster is illustrated with 6 rows, where $r_1$ and $r_2$ are the seed rows that form the template $\rho _12$; and the rationale behind exclusion of three rows and eventual inclusion of a row $(r_3)$ in the new bicluster, is explained

Each row, $r_i=\{b_{i1},\ldots ,b_{iM}\}$, is first transformed to decimal form by converting every four-bit consecutive binary number to decimal number. So each row becomes ${\hat{r}}_i=\{d_{i1},\ldots ,d_{iP} \}$,where $,P=M/4$ and $d_{ij} \in D; D=\{0,\ldots ,15\}.$ Now we define $V=\{V_{i}\}, \forall i \in D,$ where $V_0=\{0\}$ and $V_i=\{x| [[i \bigwedge x]]=x\},\forall i, x=1,\ldots ,15$ (see Fig. 2a for details). For example, in case of $V_5=\{1,4,5\}$, all binary equivalent of decimal numbers in *D* having 2nd and/or 4th bit (from right) as 0 are included in $V_5$. Note that, binary equivalent of 5 is 0101 and bit-wise AND $(\bigwedge )$ with all other binary combinations whose 2nd and/or 4th bits as 0 are 0001, 0100 and 0101, i.e., 1,4 and 5 in decimal number system. So, from definition, $V_i$ contains those non-zero decimal numbers (x) whose four-bit binary numbers return same number x after bit-wise $\bigwedge$ operation with i.

### Searching strategy

Let us assume that the seed row pair ${\hat{r}}_i$ and ${\hat{r}}_j$ creates a bicluster $B_{new}=\{I_{new},J_{new}\}$, where, $({\hat{r}}_i, {\hat{r}}_j ) \in {I_{new}}$ and $(j>i)$, as a result of applying the operator $\bigwedge$. We can also write, $B_{new}=\{I_{new},J_{new}\} =\{\{{\hat{r}}_i, {\hat{r}}_j\},\{\rho _{ij}\}\},$ where $\rho _({ij})$ is new *template* having number of nonzero terms greater than equals to $r_{min}$ and $\rho _{ij} = \bigcup _{k=0}^{P-1}[[d_{ik} \bigwedge d_{jk}]]$; where, $P=M/4$. Let $\Vert \rho _{ij} \Vert$ be the count of number of non-zero columns in $\rho _{ij}$. Note that, we proceed only if $\rho _{ij}$ is unique (new) and $\Vert \rho _{ij} \Vert \ge c_{min}$.

Now, for each of the remaining rows $({\hat{r}}_l$), in $I_{new}=R-\{{\hat{r}}_i, {\hat{r}}_j\},$ a decision is to be taken on the homogeneity criterion on, *whether or not to retain* ${\hat{r}}_l$ in $I_{new}$. This is an exhaustive checking, and usually requires bit-wise AND operations to compare every row with the *template*. In our work, we have proposed a novel mechanism by which we can column-wise search the content of every row in the set *V*, and decide *whether or not to retain* the row under consideration. If any column of a row fails the inclusion-condition, the complete row is excluded from the subsequent checking. More specifically, let $d_{kl}$ be the *k*th column decimal value of ${\hat{r}}_l$, then the row ${\hat{r}}_l$ is excluded from $I_{new}$ if $d_{k\rho } \notin V_{d_{kl}}$ (please refer to Fig. 2b for illustration). Finally, if all the columns of any given row pass the inclusion-condition, then the row is included in $I_{new}.$ We refer it as new bicluster $B_{new}=\{\{I_{new} \} \cup \{({\hat{r}}_i, {\hat{r}}_j \},J_{new} \}=\{\{I_{new}\} \cup \ \{({\hat{r}}_i, {\hat{r}}_j \},{\rho _{ij} }\}$ if and only if $|{I_{new}} \cup \{{\hat{r}}_i, {\hat{r}}_j \} |\ge r_{min}$. This procedure is continued for other all possible pair of seed rows to generate all possible maximal biclusters. Please refer to the complete Algorithm 1 for details.
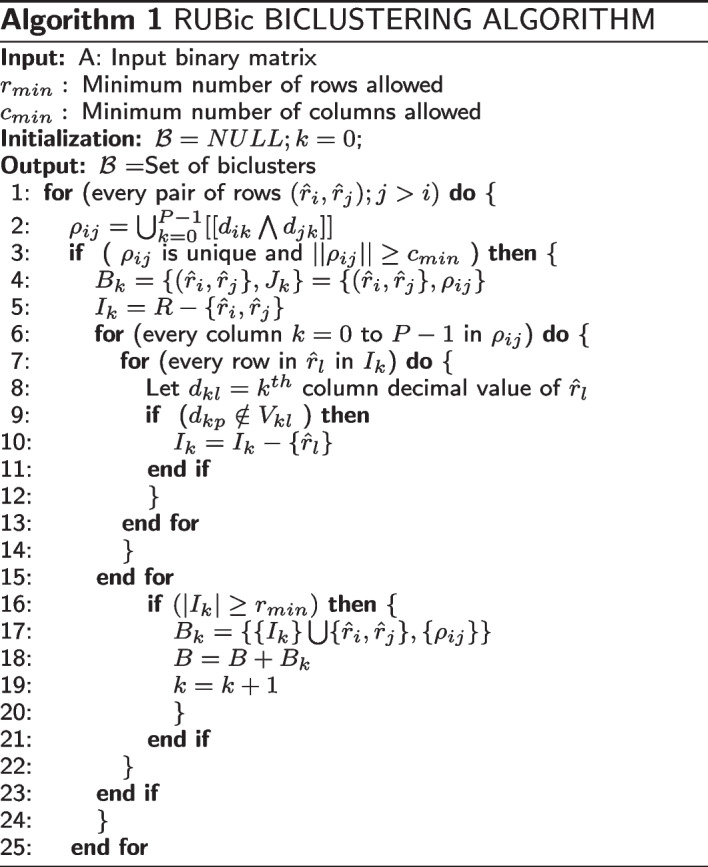


### Computational complexity

To assess the computational complexity of the newly developed RUBic algorithm, we have observed that the computational overhead is maximum during the searching process. Once we compute the template, we need to search the selected column(s) of every other row for its possible inclusion in the bicluster. Let us assume that for the 1st column of the template, *N* comparisons are required to select the row(s) and only $K_{1}$ rows are not selected due to above mentioned constraint (see Fig. 2a, b and Algorithm 1). Consequently we can exclude $K_{1}$ rows in 1st column comparison, then only $(N-K_{1})$ rows are selected for 2nd column comparison. Likewise, if $K_{2}$ rows are excluded during 2nd column comparison, then $(N-K_{1}-K_{2})$ rows are to be processed during 3rd column comparison. This procedure is continued up to the *M*th column, where ($N-K_{1}-K_{2}-\cdots -K_{(M-1)}$ ) comparisons are required to select the row(s) in the last step. Therefore, total number of comparisons which are required for the selection of rows for one bicluster can be formulated as:

1 $$\begin{aligned} T_{general}= & {} N + (N-K_{1}) + (N-K_{1}-K_{2}) + \cdots +(N-K_{1}-K_{2}-K_{M-1})\nonumber \\= & {} \sum _{i=1}^{M}N - ( \sum _{i=1}^{M-1}K_{1} + \sum _{i=1}^{M-2}K_{2} +\cdots \sum _{i=1}^{2}K_{M-2} + K_{M-1} )\nonumber \\= & {} M \times N - \{K_{1}\times (M-1)+K_{2}\times (M-2)+\cdots +2K_{M-2}+K_{M-1}\}\nonumber \\= & {} M \times N - M \times ( K_{1} + K_{2} + \cdots + K_{M-1}) - (K_{1} + K_{2} + \cdots + K_{M-1})_{c} \nonumber \\= & {} M \times (N-(K_{1} + K_{2} + \cdots + K_{M-1}))-(K_{1} + K_{2} + \cdots + K_{M-1}) \nonumber \\= & {} M \times (N-C) - C \nonumber \\ \end{aligned}$$

where $C=( K_{1}+K_{2}+\cdots +K_{(M-1)}$) is a large constant and *M* is number of columns and *N* is number of rows. Now let us derive the *best case* complexity where in 1st column comparison, all the rows except one are excluded as per our designed criteria. So, in first step, it takes *N* comparisons and in each remaining $(M-1)$ steps there is only requirement of one comparison. So total number of comparison required in best case is: $T_{best}=N+(M-1).$ In the *worst case* scenario, every column of each row satisfies our designed criteria. So it requires maximum $T_{worst}=N \times M$ number of comparisons in the worst case.

However, for the *average case*, the probability $(P_i)$ of successful search for each decimal number $(0, \ldots ,15)$ of any row in *V* is different. For (0, 15) this probability, $P_0=P_{15}=\frac{1}{16}$, because they occur only once in their respective sets $V_0$ and $V_{15}$. For the numbers 1, 2, 4 and 8, $P_1=P_2=P_4=P_8=\frac{8}{16}$. Likewise, $P_3=P_5=P_6=P_9=P_{10}=P_{12}=\frac{4}{16}$ and finally, $P_7=P_{11}=P_{13}=P_{14}=\frac{2}{16}$. Therefore, the probability of successful search of any number $(0 \ldots 15)$ of any row in *V* may be estimated as: $\frac{1}{16} \sum _{(i=0)}^{15}P_i =\frac{66}{16 \times 16} \cong 1/4.$

So, on average, in each column comparison, 25% of the rows are selected from analyses in subsequent columns. Therefore, if *N* comparisons are required in the 1st column comparison, $\frac{N}{4}$ comparisons are required in the 2nd column, $\frac{N}{4^{2}}$ comparisons in the 3rd column and so on. Therefore, total number of *average case* comparisons $T_{average}$ is estimated as follows:

2 $$\begin{aligned} T_{average}= \,& {} N+\frac{N}{4}+\frac{N}{4^2} + \cdots + \frac{N}{4^k} + C^{'}; \{ 4^{k} \le N\}\nonumber \\= \,& {} N \times \frac{1-\frac{1}{4^k}}{1-\frac{1}{4}} + C^{'}\nonumber \\= \,& {} N \times \lim _{k \rightarrow \infty } \frac{1-\frac{1}{4^k}}{1-\frac{1}{4}} + C^{'} \nonumber \\ \end{aligned}$$

where $C^{'}$ is a constant and *k* is sufficiently large. From the above formulation we can say the *average case* complexity of the developed RUBic algorithm is *O*(*N*), i.e., linearly proportional with the number of rows in the input vector.

## Experimental results

To assess the performance of the developed RUBic algorithm, we have evaluated its behaviour in terms of the execution time and quality of the biclusters generated on both synthetic and experimental biological datasets. Our biclustering algorithm is able to execute in two modes, *base-mode* and *flex-mode* depending on the two basic parameters and an additional filtering by symmetric clusters removal. At *base-mode*, we set the $r_{min}=2$ and $c_{min}=2$ to extract optimal clustering which provides maximal biclusters. We have executed our *base-mode* RUBic on the synthetic data and shows the performance improvement with *state-of-the-art*. However, we have evaluated our algorithm with two biological datasets in *flex-mode* on (a) protein–protein interaction dataset and (b) real gene expression dataset.

### Synthetic data analysis

In the current experiment, three sets of synthetic datasets were used to assess the performance of the developed algorithm and compare it with the existing *state-of-the-art* methods. We compared the performance of the base-mode RUBic with the existing fast model BiBit, as both generate maximal clusters. However, we excluded the very recent methods ARBic and QUBIC2 from this comparison as they are not designed for maximal cluster generation. Moreover, ARBic and QUBIC2 are unable to execute with $r_{min}=2$ and $c_{min}=2$ values, which causes inconsistency in clustering.

In the first experiment, synthetic binary matrices of size $200\times 200$ are used from the BiBit data repository (*Match*_*score*_*density*_200$\times 200$_*csv*) [24], with varying density and overlapping characteristics. There are ten groups of $200 \times 200$ matrices in which each group contains 10 matrices with varying density of 1’s from 5 to 50% with step wise increment of 5%. We have evaluated the average number of extracted biclusters in these ten groups of matrices and also estimated the average time of execution in each ten groups. In our experimental setup, the RUBic algorithm took $\sim 71.1$ s to process 100 synthetic matrices of this dataset, whereas BiBit [24] took $\sim 3.4$ h to complete the same task (see Table 1). It may be noted that the average time taken to extract a bicluster is decreasing with increasing density, in case of RUBic. RUBic takes 0.29 ms on average over all the bicluster produced whereas BiBit [24] takes 21.23 ms for the same purpose. Altogether, RUBic generated exactly the same number of biclusters in comparison to the 494, 872 biclusters generated by BiBit [24]. We have also evaluated the cluster quality by estimating the match scores [23] between the biclusters generated by the two methods. The average of maximum match scores of all biclusters generated by RUBic with respect to the BiBit [24] and vice-versa are exactly the same. Overall, same biclusters are extracted by RUBic, in much lesser time. Detailed results on this synthetic dataset are compiled in the Table 1.

**Table 1:** The performance of RUBic and BiBit was evaluated over the synthetic dataset with varying density of different 1’s (MATCH_SCORE_DENSITY_200X200_CSV)

Group number	Density of 1s’	Avg. ET (ms)		Avg. bicluster number		Avg. match score		Avg.ET/ bicluster (ms)	
		RUBic	BiBit	RUBic	BiBit	RUBic	BiBit	RUBic	BiBit
1	5	22	480	29	29	1	1	0.755172	16.55172
2	10	51	1609	83	83	1	1	0.613253	19.38916
3	15	97	4912	249	249	1	1	0.390361	19.72771
4	20	163	12,230	634.2	634.2	1	1	0.257332	19.28445
5	25	261	27,539	1429.3	1429.3	1	1	0.182327	19.26754
6	30	476	66,204	3294.1	3294.1	1	1	0.144592	20.09769
7	35	839	126,517	6070.4	6070.4	1	1	0.138162	20.84158
8	40	1206	219,214	9472.3	9472.3	1	1	0.127297	23.14266
9	45	1620	320,150	12,977.7	12,977.7	1	1	0.124814	24.66927
10	50	2376	446,627	15,248.2	15,248.2	1	1	0.155848	29.2905

*ET* execution time (in ms)

**Fig. 3:**
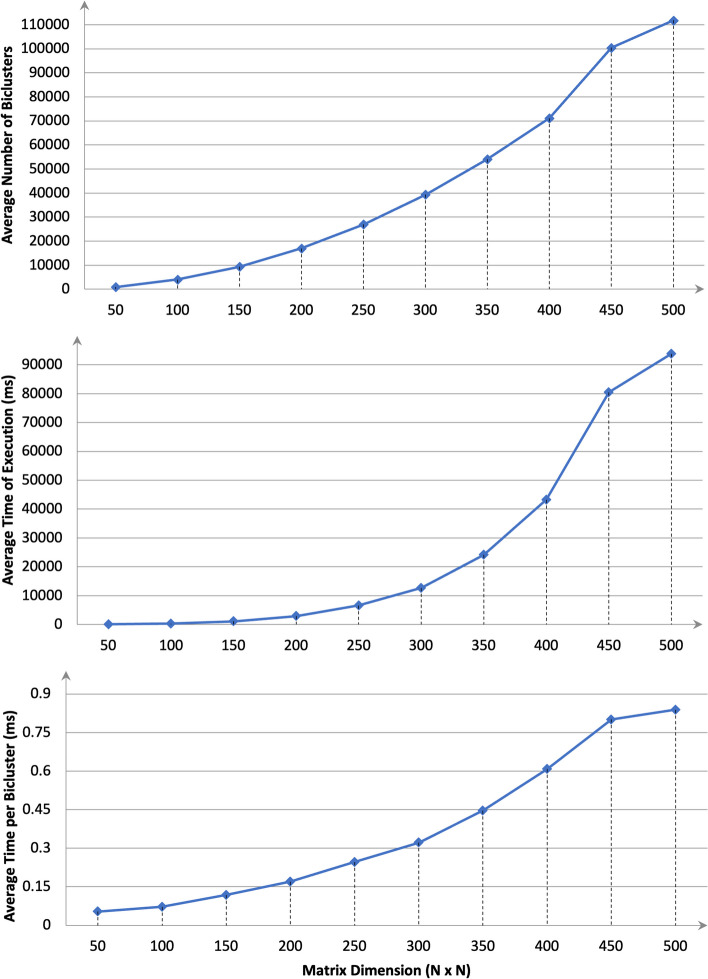
Experimental results of RUBic on 10 groups of matrices of dimension N $\times$ N. Each group contains 10 matrices with density of 1 s’ varying from 10 to 100 with step of 10%. **a** Average number of biclusters extracted, **b** average time of execution, **c** average time per bicluster

In the second synthetic data setup, to examine the scalability of the developed algorithm, we have worked over ten groups of matrices from size $50\times 50$ to $500\times 500$ with an increase of 50 rows and 50 columns in each group. Each group is having 10 matrices with the density of 1’s varying from 10 to 100% with an increment of 10% in each step. This dataset is also taken from the BiBit data repository (*Performance_test_csv*) [24] and the performance of the RUBic method over these ten groups of matrices is shown in Fig. 3. Table 2 shows the detailed results on the average time of execution over these ten groups of matrices. We can observe how the number of biclusters varies over these ten groups as the dimensionality increases. We have also plotted the average time of execution per bicluster over these groups to give an idea of the performance variation of RUBic with respect to scalability.

**Table 2:** The performance of RUBic was evaluated over the synthetic datasets with varying dimensions (PERFORMANCE_TEST_CSV)

Group number	Matrix dimension	Avg. ET (ms)	Avg no. of biclusters	Avg. ET/bicluster (ms)
1	50 $\times$ 50	49.9	925.5	0.053917
2	100 $\times$ 100	289.2	4026.3	0.071828
3	150 $\times$ 150	1101.6	9339.2	0.117954
4	200 $\times$ 200	2882.5	16,960.2	0.169957
5	250 $\times$ 250	6631.7	26,967.1	0.245918
6	300 $\times$ 300	12,670.4	39,366.9	0.321854
7	350 $\times$ 350	24,157.6	54,098.3	0.44655
8	400 $\times$ 400	43,243.3	71,116	0.608067
9	450 $\times$ 450	80,461.4	100,475.9	0.800803
10	500 $\times$ 500	93,831.5	111,868.9	0.838763

*ET* execution time (in ms)

In the third category of synthetic dataset (*Match_score_csv*) [24], 11 different matrices of sizes $100\times 100$ to $110\times 110$ with varying degree of overlapping are implanted; ground-truth biclusters of size $10\times 10$ to $20\times 20$ are used. We have used the *average bicluster relevance* which measures up to what level the mined biclusters represent ‘true’ biclusters which we have already introduced in the dataset. We have used the *average module recovery* to know how far each of the true biclusters is recovered. Both of the measures are normalized between (0, 1), where higher value reveals that the set of generated biclusters is same as to true set of biclusters and 0 represents that the sets are disjoint [23]. The objective of this experiment was to evaluate average bicluster relevance and module recovery of the two methods, RUBic and BiBit [24], under consideration. Table 3 shows that both the methods extract the exactly the same biclusters with similar bicluster relevance and module recovery scores.

**Table 3:** The performance of RUBic and BiBit was evaluated over implanted biclusters of varying sizes (10 $\times$ 10 to 20 $\times$ 20) with different overlapping degrees on (MATCH_SCORE_CSV)

Group number	Overlapping degree (%)	Matrix size	Avg. bicluster relevance		Avg. module recovery	
			RUBic	BiBit	RUBic	BiBit
1	0	100 $\times$ 100	1	1	1	1
2	1	101 $\times$ 101	1	1	1	1
3	2	102 $\times$ 102	0.586039	0.586039	1	1
4	3	103 $\times$ 103	0.612996	0.612996	1	1
5	4	104 $\times$ 104	0.63648	0.63648	1	1
6	5	105 $\times$ 105	0.657143	0.657143	1	1
7	6	106 $\times$ 106	0.675481	0.675481	1	1
8	7	107 $\times$ 107	0.691877	0.691877	1	1
9	8	108 $\times$ 108	0.702873	0.702873	0.989474	0.989474
10	9	109 $\times$ 109	0.716418	0.716418	0.99	0.99
11	10	110 $\times$ 110	0.728741	0.728741	0.990476	0.990476

### Biological data analysis

We have also assessed the performance of the RUBic method on the latest biological datasets, the *Homo sapiens* protein–protein interaction (PPI) dataset [29]. Initially it contained 6, 247 number of protein–protein interactions. We have removed self-interactions from the dataset which reduces it to 5823 number of interactions only. However, it only contains 4085 number of unique proteins and we mapped it into a binary matrix of size $4085\times 4085$ where 1’s represent interaction and 0’s represent non-interaction among the corresponding proteins. We have extracted the data for extracting biclusters with minimum number of row as 2 and minimum number of column as 2. We have observed that our method RUBic generates 1840 maximal biclusters in $\sim 48.6$ s. Then the same dataset is used for BiBit [24] and it generates the same 1, 840 number of biclusters in $\sim 251.6$ s. We have also worked over the central nervous system (CNS) embryonic tumor gene expression dataset [30] of size $7129 \times 40$ were considered. We have also executed both modes of RUBic (base and flex). In this biological dataset, RUBic-base generates 747,069 maximal biclusters in $\sim 101$ min, whereas, BiBit [24] produces the same number of biclusters in $\sim 56$ h (see Table 4).

**Table 4:** The performance of RUBic and BiBit was evaluated over a protein–protein interaction (PPI) dataset of Homo sapiens and a Central Nervous System (CNS) embryonic tumor gene expression dataset

Dataset	Methods	Execution time (ms)	# of biclusters
Human PPI	BiBit	251,652	1840
	RUBic-base	48,588	1840
	RUBic-flex	32,156	131
	ARBic	1,055,457	204
	QUBIC2	32,789	189
CNS	BiBit	202,308,623	747,069
	RUBic-base	6,066,520	747,069
	RUBic-flex	9804	1069
	ARBic	68,920	132
	QUBIC2	68,137	1508

### Performance on expression datasets

**Fig. 4:**
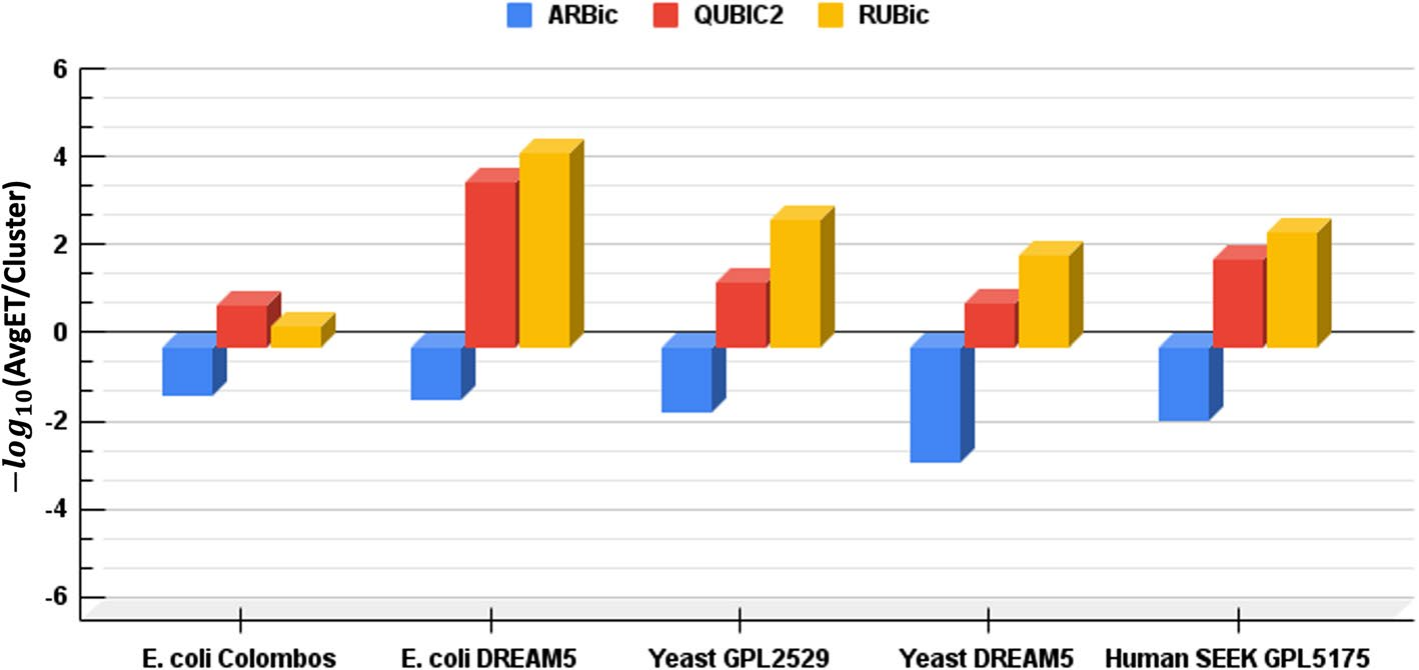
Average execution time ($-{log}_{10}({AvgET/cl})$) per cluster on five real datasets

To compare RUBic with other algorithms on real datasets with a large number of columns ($>500$), we evaluated five datasets from E. coli, yeast, and human tissues. To ensure consistency between the algorithms, we conducted experiments using RUBic-flex algorithm with two parameter ($r_{min}$, $c_{min}$) values synchronized with both ARBic and QUBIC2. We have executed all the algorithms using the optimal parameters specified in their respective publications on these five datasets [30]. First, all the gene expression values are discretized into binary matrix using Mixture of Left Truncated Gaussian Distributions model as described in [26]. On these binary patters, RUBic-flex is employed to extract the significant biclusters. It has been observed that in all 5 datasets, RUBic-flex shown a significant performance improvement in terms of total execution time speed-up (see Table 5) and average time per clusters compared to ARBic and QUBIC2. However, in average time per cluster, RUBic also surpass ARBic and QUBIC2 in all datasets with only exception of *E. coli* Colombos as depicted in Fig. 4. In all these datasets, ARBic generates significantly less number of clusters, where in *E. coli* Colombos and Yeast DREAM5 dataset, RUBic produces less clusters compared to QUBIC2 and rest three datasets RUBic generates higher number of clusters with less execution time. The detailed statistics of generated clusters and corresponding execution time (ET) are reported in Table 5.

**Table 5:** Significance speed up of RUBic on five real datasets measured in terms of execution time (s)

Dataset	Genes	Conditions	ARBic		QUBIC2		RUBic	
			Cls	ET	Cls	ET	Cls	ET
*E. coli* colombos	2093	2470	267	3215.451	2869	319.55	598	210.41
*E. coli* DREAM5	2442	805	137	2143.9128	2821	207.83	3163	145.337
Yeast GPL2529	3178	3025	415	12297.099	3555	1276.55	5668	752.15
Yeast DREAM5	3292	536	36	14524.4534	3311	1660.93	3163	740.66
Human SEEK GPL5175	4436	2308	296	13954.656	5044	1278.3	6215	1017.81

*Cls* number of clusters, *ET* execution time (in s)

**Fig. 5:**
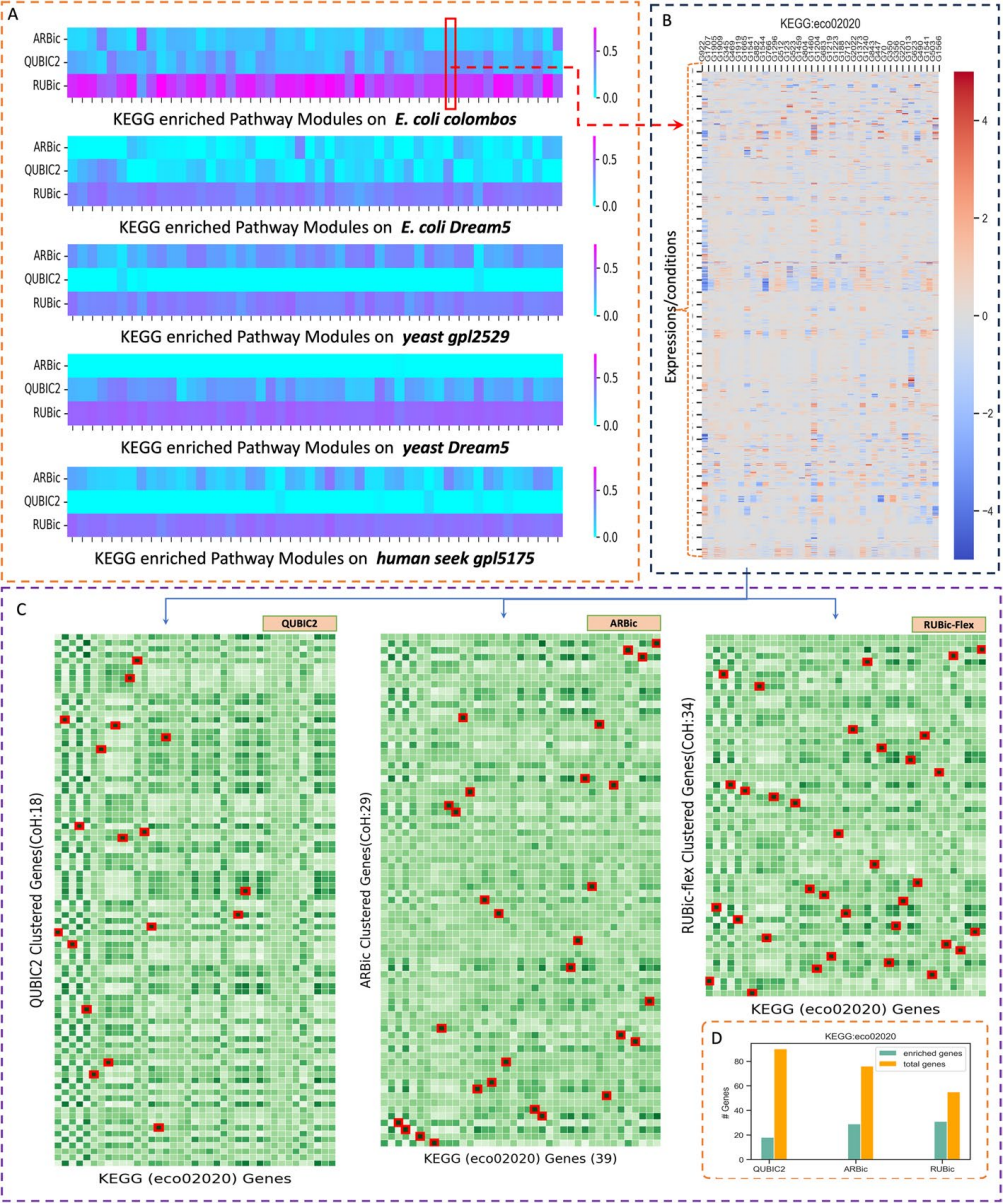
Significant match comparison with respect to KEGG annotated clusters. **A** The heatmap columns represents KEGG pathway annotations and row represents biclustering. Each cell represents average matching scores of top 50 biclustering solutions with respect to KEGG enriched clusters. **B** Heatmap for reference KEGG clusters with expression values. **C** Represents the heatmap for expression level correlation matrix from resultant biclusters of RUBic-Flex, QUBIC2 and ARBic. *CoH* correlation hits, indicated number of common genes between KEGG and other cluster solutions. Red boxed cells within the heatmap represents KEGG(eceo2020) enriched gene that are present in the respective clusters. **D** Barplot representation of enriched gene count in each clustering approach and total number of genes within clusters

As the true biclusters in real datasets are unknown, we evaluated each bicluster identified by each algorithm by using KEGG [28] biological pathway-based analysis on *E. coli* Colombos to demonstrate the efficacy of our proposed approach, RUBic. To evaluate the significance of our algorithm, we incorporated all the pathways as compared to ARBic. We found that the match score of each cluster with the KEGG enriched pathway clusters was similar for all three approaches, ARBic, QUBIC2, and RUBic. The heatmap representation of the match score per KEGG enriched cluster is shown in Fig. 5A. In most of the pathways, based gene annotations are well-mapped with the biclusters found in RUBic. Each cell represents average matching scores of top 50 biclustering solutions with respect to KEGG enriched clusters (see Fig. 5A).

For example, in the *ecoli_colombos* dataset for the KEGG pathway‘Two-component system’ *eco02020* (see expression heatmap Fig. 5B), we found a significant number of overlapping genes (34) with RUBic clusters where in QUBIC2 and ARBic extracts 18 and 29 enriched genes (correlation hits), respectively with a maximal matching cluster. Figure 5C represents the heatmap for the expression level correlation matrix from resulting biclusters of RUBic-Flex, QUBIC2 and ARBic. Red-boxed cells within the heatmap represent KEGG(*eco02020*) enriched genes present in the respective clusters, whereas Fig. 5D shows the enriched gene count in each clustering approach and the total number of genes within those clusters.

## Conclusion

In this work we propose a novel algorithm for fast extraction of biclusters from binary datasets. We have evaluated its performance on both synthetic and biological datasets and compared the results with the existing *state-of-the-art*. We also estimated the best case, worst case, and average case complexity of the developed method and attempt to show that the average case computational complexity is almost linearly proportional to the increase in the size of the dataset. Our approach can operate in two modes: base-mode and flex-mode, resulting in two types of clustering solutions - maximal biclusters and biologically plausible biclusters.

The performance evaluation was carried out on three carefully selected benchmark synthetic datasets using base-mode and flex-mode to assess the effects of varying density, degree of overlap, and dimensionality on the developed method. Two biological datasets were also considered. The first one was the human PPI dataset of low density of interactions in a large square matrix $4085 \times 4085$ used to assess the performance of the method. The second dataset was a large CNS gene expression data used to test the performance of the method. In the case of the CNS gene expression dataset, RUBic generated a large number of biclusters in less than 2 h, which took $\sim 56$ h by the BiBit algorithm [24]. This observation highlights the robustness of our method with respect to the scalability of the dataset.

The performance of RUBic was also evaluated on five gene expression datasets, two from *E. coli*, two from yeast, and one from human tissues. Our RUBic-flex mode biclustering showed a significant performance improvement in terms of total execution time speed-up compared to the most recent and popular biclustering strategies ARBic and QUBIC2. Finally, the extracted clusters were evaluated and validated with match scores of resultant clusters with KEGG enriched pathway clusters for biological significance.

Thus, not only is our RUBic algorithm faster than its competitors, it also produces an optimal set of biologically relevant biclusters, and established a new state of the art in across several benchmark synthetic, PPI and gene expression datasets, and hence is expected to have generalised applications across proteomic and genomic interactions, including high impact tasks of automated drug repurposing and drug discovery.

## Data Availability

The different public datasets used in this work are referred to in their respective publications, as described in the "Biological data analysis" Sect. .

## Code availability

The code is available at (https://github.com/CMATERJU-BIOINFO/RUBic) for academic use only.
